# Migrant breast cancer patients and their participation in genetic counseling: results from a registry-based study

**DOI:** 10.1007/s10689-016-9871-y

**Published:** 2016-02-01

**Authors:** J. E. Baars, A. M. van Dulmen, M. E. Velthuizen, E. B. M. Theunissen, B. C. Vrouenraets, A. N. Kimmings, T. van Dalen, B. van Ooijen, A. J. Witkamp, M. A. van der Aa, M. G. E. M. Ausems

**Affiliations:** Division of Biomedical Genetics, Department of Medical Genetics, University Medical Center Utrecht, PO Box 85090, 3508 AB Utrecht, The Netherlands; NIVEL (Netherlands Institute for Health Services Research), Utrecht, The Netherlands; Department of Primary and Community Care, Radboud University Medical Center, Nijmegen, The Netherlands; Faculty of Health Sciences, Buskerud and Vestfold University College, Drammen, Norway; Division of Surgery, St. Antonius Hospital, Nieuwegein, The Netherlands; Division of Surgery, St. Lucas Andreas Hospital, Amsterdam, The Netherlands; Division of Surgery, Slotervaart Hospital, Amsterdam, The Netherlands; Division of Surgery, Diakonessen Hospital, Utrecht, The Netherlands; Division of Surgery, Meander Medical Center, Amersfoort, The Netherlands; Division of Surgery, University Medical Center Utrecht, Utrecht, The Netherlands; Comprehensive Cancer Centre the Netherlands (IKNL), Utrecht, The Netherlands

**Keywords:** Cancer, Oncology, Genetic testing, Breast, Moroccan, Turkish

## Abstract

Certain ethnic groups seem to have less access to cancer genetic counseling. Our study was to investigate the participation in cancer genetic counseling among migrant breast cancer patients of Turkish and Moroccan origin. Hospital medical records of Turkish and Moroccan and of a comparative group of non-Turkish/Moroccan newly diagnosed breast cancer patients were studied. All women were diagnosed between 2007 and 2012. Eligibility for genetic counseling was assessed with a checklist. A total of 156 Turkish/Moroccan patients were identified, and 321 patients were assigned to the comparative group. About one third (35 %) of the Turkish/Moroccan patients fulfilled criteria for breast cancer genetic counseling, compared to 21 % of the comparative group (*P* = 0.001); this was largely due to a relatively young age at diagnosis in the migrant group (26 % <40 years vs 5 % in the comparative group, *P* = 0.0001). Uptake of genetic counseling among eligible patients was 47 % in the migrant group and 56 % in the comparative group; differences in uptake were seen among the patients diagnosed before 40 years of age (48 % in the migrant group vs 81 % in the comparative group; *P* = 0.021). When adjusted for age at diagnosis, ethnicity was associated with discussing referral to genetic counseling and its actual uptake. The Turkish/Moroccan ethnicity appears to be associated with a lower uptake of genetic counseling, mainly caused by the lower uptake in the young age-group. The major barrier to participation in genetic counseling seems to lie within the referral process.

## Introduction

In general, a BRCA mutation is detected in about 10 % of the tested breast cancer patients [1]. Because younger women diagnosed with breast cancer and those with a family history of breast and/or ovarian cancer are at higher risk of carrying a BRCA1/2 gene mutation, diagnostic DNA testing is offered to this subset of breast cancer patients [2].

Once these patients are referred by their physicians to family cancer clinics they can opt for genetic counseling and testing (GCT). During GCT genetic counselors give information about the risk to develop (a second) cancer for the patient and her relatives and about options for cancer prevention and early detection [3].

Hall and coworkers [4] showed that the prevalence of BRCA1/2 mutations is high and nearly identical across different ethnicities, not only in Caucasian women. However, testing volumes were disproportionately low among women from non-European ancestry [4]. Others also observed a low uptake of GCT among ethnic minorities, i.e. Afro-Americans and Hispanics in the US [5–8].

Known barriers towards the use of GCT were socioeconomic barriers (e.g., time, access, costs, geographic, awareness, language and cultural) and psychosocial barriers (e.g., inaccurate cancer risk perception, medical mistrust and perceived disadvantages to genetic services) [6, 9, 10].

Also in the Netherlands, patients from non-Western descent seem to be underrepresented in cancer GCT [11]. At present, in the Netherlands nearly two million (12 %) of the population have a non-Western background [12]. About 40 % of the inhabitants from non-Western descent are from Turkish (20 %) and Moroccan (19 %) origin [12], which makes them the largest migrant groups in the Netherlands. The first generation of these mostly low educated migrant groups is known to have major language difficulties [13], which may hamper their access to health care. Although breast cancer among Turkish and Moroccan migrant women is less prevalent than among native women [14, 15], higher relative excess mortality from breast cancer in these migrant women might point toward inadequate access to health care and treatment in this group [14].

In Turkey and Morocco, the countries of origin, studies addressing GCT among breast cancer patients are yet upcoming and mainly focus on the prevalence of BRCA mutations [16–20]. However, these studies do not reflect on referral rates and participation in GCT, which is as yet unknown. In many Arabic cultures and countries of the Greater Middle East, a cancer diagnosis is still accompanied with social stigma and misperceptions regarding the incurability of the disease [21], which could hinder the participation in breast cancer genetic counseling.

The aim of the present study is to investigate the participation of Turkish and Moroccan patients in breast cancer GCT. More specifically, our research questions include: (1) What proportion of Turkish and Moroccan breast cancer patients fulfils criteria for referral for GCT and what is the actual uptake?; (2) Does eligibility and uptake differ from non-Turkish/Moroccan patients?; (3) Is ethnicity associated with discussing GCT referral and uptake of GCT?

## Materials and methods

### Sample

The study population included a Moroccan and Turkish patient group and a non-Turkish/Moroccan patient group. These patients were diagnosed with breast cancer between January 2007 and December 2012 in six hospitals in the Utrecht region and in Amsterdam, the Netherlands.

### Procedure

Medical records of female breast cancer patients were studied between April 2013 and April 2014. Patients from Turkish and Moroccan descent were identified by a name-based approach. This approach has been used in different scientific studies among Turks and Moroccans in the Netherlands and Germany [15, 22–25]. If information about the country of birth was available in the medical records, Moroccan/Turkish ethnicity was checked and registered. Because our pilot study among Turkish and Moroccan breast cancer patients showed a higher eligibility to genetic counseling compared to the average Dutch breast cancer patient population, a twice as large comparative group of the remaining non-Turkish and non-Moroccan breast cancer patients was randomly selected by SPSS per hospital, to get an about equally large comparison group (see “Appendix”). The medical records were studied by JB and MV, the first 20 checklists were filled in by both researchers together. A random sample of 10 % of the checklists were filled in by both researchers, which showed an interrater reliability of 0.94 (Cohen’s kappa) on the variables shown in Table 1.

**Table 1:** Checklist criteria for eligibility to be referred to breast cancer genetic counselling

One or more of the following
Breast cancer diagnosis <40 years of age
Breast cancer diagnosis <50 years of age and
Bilateral breast cancer (synchronic or metachronic)
First degree relative with breast cancer
Breast cancer diagnosis at any age and
Known BRCA1/2 mutation in the family*
Personal history of contralateral breast cancer <50 years
Personal history of ipsilateral breast cancer <50 years*
Personal history of ovarian cancer*
Relative with ovarian cancer in the same part of the family
Male relative with breast cancer in the same part of the family*
Two or more first/second degree relatives with breast cancer in the same part of the family

* Not included in the overall Cohen’s Kappa, because the variable is a constant (and 100 % agreement)

### Variables

Data extracted from the medical records with a checklist included variables to assess the eligibility for GCT, such as age at diagnosis and characteristics of the patient and the family history, if present (see Table 1). Because the patients were diagnosed in the years 2007–2012, the eligibility criteria were based on the national guidelines of that time [26], except for early age at diagnosis (e.g. before 40 years of age was common practice). Other variables to be noted were: ethnicity, year of birth, type of surgery, TNM stage, and whether language problems were reported and an interpreter was present during consultations. Finally, we assessed ‘discussing GCT referral’ which represents whether or not referral to GCT was discussed with the patient and reported in the medical file, and the actual ‘uptake’ of GCT. In case a discussion with the patient about GCT was not reported in the medical record, but GCT was performed (uptake was positive), patients were automatically coded as ‘having discussed GCT referral’.

Information in the medical records about the family history was coded into three categories; (1) a *complete* family history: information about the occurrence or absence of *breast and ovarian cancer* in the family was present; (2) a *‘partial’* family history: only information about family history of *breast cancer*, and (3) ‘*no information’*: no information about the family history of breast and ovarian cancer.

### Analysis

Differences between groups were analysed with Chi square and Student’s *t* tests. Furthermore, to answer the third research question (to identify determinants for the uptake of genetic counseling and to explore whether ethnicity is associated with GCT uptake), a logistic regression analysis was performed within those eligible for genetic counseling in the total sample. Independent variables included ethnicity (Turkish/Moroccan versus non-Turkish/Moroccan); furthermore, potential determinants to be related to the outcome variable (i.e. age at diagnosis, TNM-stage, language difficulties, criteria for discussing GCT referral to genetic counseling (e.g. family history, year of diagnosis, see Table 2) were univariately tested and if correlated with the outcome variables (*P* < 0.05), they were included in the logistic regression analysis.

**Table 2:** Characteristics of breast cancer patients diagnosed 2007–2012*

	Turkish N = 55 n (%)	Moroccan N = 101 n (%)	*P*	Total Turkish/Moroccan N = 156 n (%)	Comparative group N = 321 n (%)	*P*
Socio-demographic and clinical variables						
Age at diagnosis mean (SD)	51.70 (12.61)	47.81 (11.21)	0.05	49.18 (11.83)	61.73 (14.62)	0.0001
Surgery						
BCT	35 (66.0)	56 (57.7)	0.32	91 (60.7)	178 (59.3)	0.79
Mastectomy	18 (34.0)	41 (42.3)		59 (39.3)	122 (40.7)	
TNM-stage						
0–I	41 (75.9)	55 (56.7)	0.02	96 (63.6)	225 (74.8)	0.01
II	3 (5.6)	20 (20.6)		23 (15.2)	45 (15.0)	
III	7 (13.0)	9 (56.2)		16 (10.6)	19 (6.3)	
IV	3 (5.6)	13 (13.4)		16 (10.6)	12 (4.0)	
Poor or no mastery of Dutch language	28 (50.9)	40 (39.6)	0.17	68 (43.6)	4 (1.2)	0.0001
Consultation translated	23 (41.8)	30 (29.7)	0.13	53 (34.0)	2 (0.6)	0.0001
By family	19 (34.5)	28 (27.7)		47 (30.1)	2 (0.6)	
≥1 time by professional	4 (7.3)	2 (2.0)		6 (3.8)	(0)	

*BCT* breast conserving therapy, *TNM* tumor, nodes, metastases, *TNM* staging takes into account the size of the tumour (T), whether the cancer has spread to the lymph nodes (N), and whether there are metastases (M)

The TNM-stages are defined from group 0 through IV, and indicate the increasing extent of disease at the time of the initial diagnosis. Stage 0 includes breast cancer in situ, Stages I–III include different tumour sizes and lymph nodes without distant metastases, and Stage IV indicates the presence of distant metastases

* Number of respondents may vary across variables according to missing data

## Results

### Socio-demographic and clinical variables

For the period 2007–2012 we identified 156 female migrant breast cancer patients (55 with a Turkish and 101 with a Moroccan background) using the name-based approach and 321 female patients were randomly assigned to the comparative group (see flowchart in “Appendix”). Patient characteristics are shown in Table 2. Statistically significant differences between groups were found for the mean age at diagnosis, and the levels of the TNM-stages. Moroccan women had a higher proportion of TNM-stage IV. In the total Turkish/Moroccan group there were less patients with a more favourable disease stage compared to the comparative group.

In 44 % of Turkish/Moroccan patient records, the records indicated that the patient had poor or no mastery of the Dutch language. In 34 % of all Turkish/Moroccan patients one or more consultations had been translated by an interpreter, mostly family-related.

### Criteria for referral to GCT

A higher proportion of the total group of Turkish/Moroccan breast cancer patients fulfilled criteria for referral to genetic counseling (35 %) as compared to the non-Turkish/Moroccan comparative group (21 %; *P* = 0.001). This difference was caused by the large portion of Turkish/Moroccan patients who were younger than 40 years of age when diagnosed (26 % in the Turkish/Moroccan group compared to 5 % in the comparative group; *P* = 0.0001; see Table 3).

**Table 3:** Criteria for referral to genetic counseling*

	Turkish N = 55 n (%)	Moroccan N = 101 n (%)	*P*	Total Turkish/Moroccan N = 156 n (%)	Comparative group N = 321 n (%)	*P*
Total eligible	18 (32.7)	37 (36.6)	0.63	55 (35.3)	66 (20.6)	0.001
Personal cancer history						
Age at diagnose						
<40 years	11 (20.0)	29 (28.7)	0.23	40 (25.6)	16 (5.0)	0.0001
Contralateral or ipsilateral BC < 50 years	2 (3.6)	3 (3.0)	–	5 (3.2)	7 (2.2)	0.503
OVCA	0	0	–	0 (0)	2 (0.6)	–
Family cancer history						
OVCA in family	0	1 (1)	–	1 (0.6)	12 (3.7)	–
BC in family	8 (14.5)	8 (7.9)	0.19	16 (10.3)	39 (12.1)	0.54
Male BC in family	1 (1.8)	2 (2.0)	–	3 (1.9)	1 (0.3)	–

*BC* breast cancer, *OVCA* ovarian cancer

Contralateral or ipsilateral BC < 50 years: *BC patients of all ages with a* contralateral and/or ipsilateral BC diagnosed before the age of 50

OVCA in family: all BC patients who have a family member with OVCA

For a few variables, Chi square statistics could not be calculated because > 20 % of the cells had an expected count of 0.5

BC in family: BC patients of all ages at diagnosis who have two or more first and/or second degree family members with BC (same family branch) and/or one first degree family member with BC diagnosed before the age of 50 and/or patients who were younger than 50 years at diagnosis who have at least one family member with BC

Male BC: 1 or more male first degree family members with BC

* Patients can fulfil one or more criteria

### Family history

The majority of patients had some data on their family history in their medical file. In the medical records of 42 % in the Turkish/Moroccan group and 39 % in the comparative group a ‘complete’ family history was found. In about half of the patients in the Turkish/Moroccan group (49 %) and in the comparative group (47 %) the information in the medical file included only information about the family history of breast cancer and not about ovarian cancer (a ‘partial’ family history). No differences between the groups were found with regard to the information on family history in the medical records (*P* = 0.23).

### Uptake of breast cancer genetic counseling and testing

Discussing the possibilities of GCT with the patient and proposing referral is a necessary step to get access to cancer genetic counseling and DNA testing. Table 4 shows the proportion of eligible patients who had discussed GCT with their physician (‘discussing GCT referral’) and the proportion of eligible patients who received GCT (‘uptake’).

**Table 4:** ‘Discussing GCT referral’ and ‘uptake’ in genetic counseling and testing

Selection of patients eligible for GCT	Turkish/Moroccan			Comparative		
	Total N	Discuss GCT referral n (%)	Uptake GCT n (%)	Total N	Discuss GCT referral n (%)	Uptake GCT n (%)
Total eligible	55	31 (56.4)	26 (47)	66	39 (59.1)	34 (56)
Age at diagnosis <40	40	22 (55.0)*	19 (47.5)†	16	13 (81.2)*	13 (81.2)†
Fam history BC/OVCA	17	12 (70.6)	11 (64.7)	48	27 (56.3)	25 (52.1)

*GCT* Genetic counselling and testing

Fam history BC/OVCA: 2 or more first and/or second degree family members with BC (same family branch) and/or a family member with history of ovarian cancer

Participants can fulfil one or more criteria as shown in Table 3; furthermore, the ‘Total eligible group’ also includes patients with contralateral or ipsilateral breast cancer <50 years who did not fulfil the criteria ‘Age at diagnosis <40-group’ nor the ‘Fam history BC/OVCA’

* *P* = 0.067

† *P* = 0.021

When looking at patients eligible for GCT, we found no statistically significant differences between both groups with regard to discussing GCT referral and uptake of GCT. When focusing on the younger patients (<40 years at diagnosis) only, a trend was observed for discussing GCT referral: with 55 % of patients in the Turkish/Moroccan group GCT had been discussed, compared to 81 % in the comparative group (*P* = 0.067). Also the actual uptake differed between the groups eligible for genetic counseling due to their young age at diagnosis (<40 years) (48 % in the Turkish/Moroccan group versus 81 % in the comparative group (*P* = 0.02).

To study whether ethnicity was associated with ‘discussing GCT referral’ and ‘uptake of GCT’, variables that were significantly correlated with discussing GCT referral and uptake of GCT were included in a logistic regression analysis, with ethnicity as one of the independent variables. When controlled for age at diagnosis, Turkish/Moroccan ethnicity was negatively associated with ‘discussing GCT’ referral and ‘uptake of GCT’ (Table 5).

**Table 5:** Adjusted odds ratios and 95 % CIs for ‘discussing GCT referral’ and BRCA1/2 testing among breast cancer patients eligible for GCT

Predictors	Discuss GCT referral	Uptake of GCT
	Adjusted OR (95 % CI)	Adjusted OR (95 % CI)
Total	N = 121	N = 121
Age at diagnosis (continuous variable)	0.92 (0.88–0.96)	0.92 (0.88–0.96)
Ethnicity		
Non-Turkish/Moroccan (ref)		
Turkish/Moroccan	0.38 (0.15–0.93)	0.28 (0.11–0.71)
Nagelkerke R square	0.21	0.22
*P* value model	0.0001	0.0001

## Discussion

Overall, the participation in GCT among eligible breast cancer patients was about the same amongst the migrant group and a comparative group of randomly selected non-Turkish/Moroccan patients. However, we found lower GCT participation rates among young Turkish/Moroccan migrant breast cancer patients (<40 years at diagnosis). When controlled for age at diagnosis, ethnicity showed to be a predictor of ‘discussing GCT referral’ as well as ‘uptake of breast cancer GCT’. Our data suggest that the major barrier to the uptake of GCT within Turkish and Moroccan migrant groups lies within the referral process. Once referred, our data shows most are likely to follow the advice of their physician. Our study showed that nearly half of the migrant breast cancer patients had major language difficulties, however this was not a predictor of lower uptake. Possibly, language barriers, for example when a consultation is translated by a family member, might be underreported in the medical files. It is as yet unknown what may contribute to a lower participation in GCT among young migrant breast cancer patients of Turkish/Moroccan origin. From the perspective of the physician, failure to identify and refer eligible women towards GCT may be related to the lack of time to assess a complete familial cancer history [27]; yet, our study showed no differences in assessing family history between the ethnic groups. We know that the Turkish and Moroccan migrant groups in the Netherlands are generally low educated [13]. From other studies on health inequalities in cancer care it seems that lower educated people are more prone to less adequate care [28]. Further study should focus on the possible barriers in the Turkish and Moroccan groups and should explore the perceptions of the physicians.

Ethnicity and the age at diagnosis were predictors of ‘discussing GCT referral’ and ‘uptake of GCT’ in a multivariate analysis. The latter seems to be the strongest predictor of discussing GCT referral and uptake of GCT in the total group eligible; the younger the patient, the more likely they are to be referred to GCT.

As shown in our study, a large proportion of Turkish and Moroccan breast cancer patients is eligible for GCT due to a young age at diagnosis (26 %). This is noteworthy, given that in the general population of invasive and non-invasive breast cancer patients in the Netherlands, only 4.4 % is younger than 40 years at time of diagnosis [29]. This proportion corresponds to our results in the comparative group. The relatively young age at diagnosis in the migrant group is in line with studies in Turkey and Morocco [30–33]. There could be a variety of factors contributing to the relatively high proportion of young migrant breast cancer patients, such as lifestyle related and reproductive factors, which are likely to be influenced by the acculturation process. Another possible explanation lies in the dissimilar age-distribution of the migrant populations in the host-country. Dutch demographics show a relatively young Turkish and Moroccan population with rather few older people as compared to the Dutch native population [34]. Further studies are needed to investigate determinants for the relatively large group of young individuals within the total migrant breast cancer patient population.

There are a number of limitations in this study including its retrospective design based on medical records, the generalizability of the results, and the relatively small sample sizes. Although it was the best available option, our name-based approach could have biased our results in the sense that we could have missed patients who have an extraordinary name. Furthermore, a name-based approach might miss patients who adopted the name of a native spouse. However, full names were checked, and Turkish and Moroccan women tend to marry within the same cultural background [35]. In our analyses we have taken the Turkish and the Moroccan patients together because these are both large migrant groups, both have language barriers, and mostly a lower social economic status. Despite the fact that they share a lot of similarities, one should be aware that these are also culturally diverse groups. With regard to the comparative group, it can not be excluded that this group contained other (Western and non-Western) migrant patients. It is possible that other migrant groups also have a low referral to and uptake of genetic counselling, and the differences between groups may therefore be under-estimated. Furthermore, a possible incompleteness of the registration in the hospital records might have resulted in an underestimation of discussing GCT referral and uptake rates. We had access to the hospital medical records of the participating six hospitals. Patients could have been referred to other hospitals for example for adjuvant radiotherapy, and these records have not been checked because they fall out of the scope of the current study. Moreover, the degree in which information such as the family pedigree was organised in the electronic medical records differed between the hospitals. Although the good interrater reliability suggests that this did not affect the quality of our data, a unified form of recording family history should be recommended to get an easier view and clearer picture of the family pedigree.

Referral for genetic counseling may contribute to early breast cancer detection and intervention. Besides preventive measures that are available for patients who test positive for a BRCA1/2 gene mutation and have increased risk to develop a second primary breast cancer and ovarian cancer [36–38], GCT also provides information for the family members of tested individuals. Also for patients with an inconclusive test result (no pathogenic BRCA gene mutation has been found), an increased breast cancer risk for relatives cannot be ruled out. During the counseling sessions, the affected—index—patients receive information such as their family members’ breast cancer risks and screening possibilities. Therefore, it is of importance that all patients eligible and willing to participate in GCT are identified. Our data suggest a lower uptake among young Turkish/Moroccan breast cancer patients, mainly due to a low referral rate among these groups. Further research is needed with regard to the possible explanations for this low referral rate.

Our study showed a relatively large group of young Turkish and Moroccan breast cancer patients diagnosed before the age of 40. Changes of lifestyle and reproduction and the ageing of the migrant groups might eventually lead to a growing proportion of cancer incidence among non-Western minorities. Insight in the GCT referral and participation rates among culturally diverse patient groups will gain importance in order to plan future counseling practices and to ensure equal access to cancer GCT.

## Figures and Tables

**Fig. 1:**
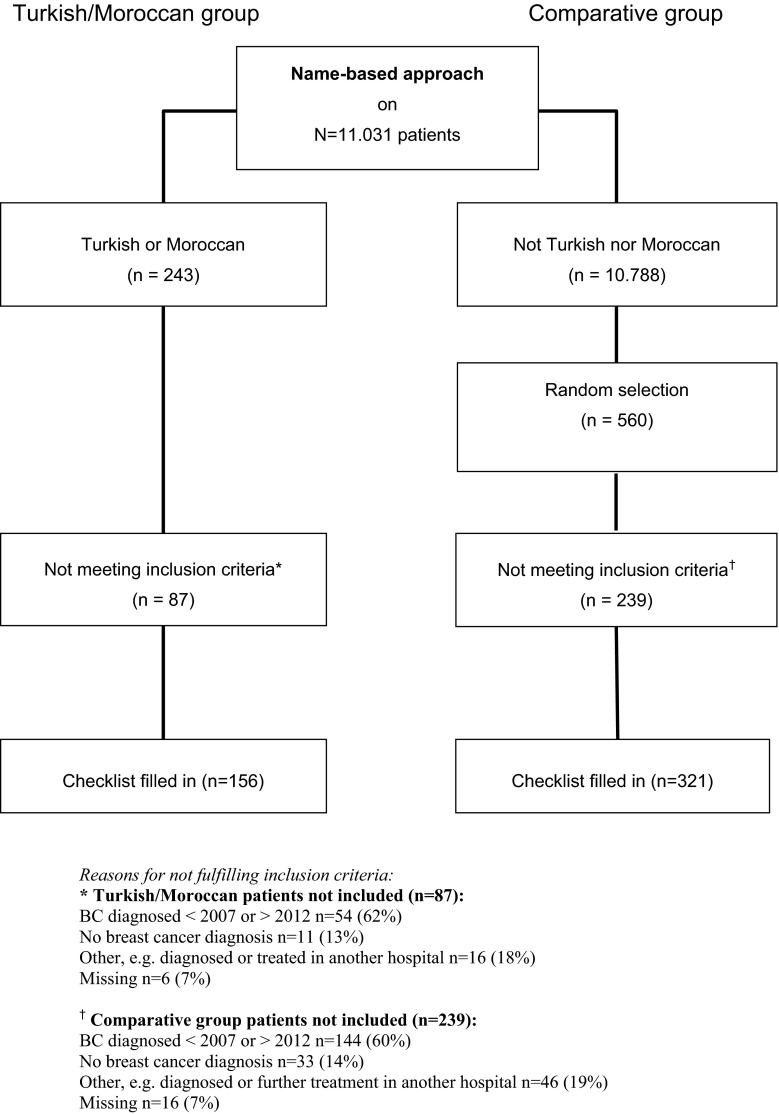
Flowchart

## Appendix

See Fig. 1.

## References

[1] Wevers MR, Aaronson NK, Verhoef S, Bleiker EM, Hahn DE, Kuenen MA, van der Sanden-Melis J, Brouwer T, Hogervorst FB, van der Luijt RB, Valdimarsdottir HB, Theunissen EB, de Roos MA, Borgstein PJ, Vrouenraets BC, Vriens E, Bouma WH, Rijna H, Vente JP, Witkamp AJ, Rutgers EJ, Ausems MG (2014). Impact of rapid genetic counselling and testing on the decision to undergo immediate or delayed prophylactic mastectomy in newly diagnosed breast cancer patients: findings from a randomised controlled trial. Br J Cancer.

[2] Bevers TB, Anderson BO, Bonaccio E, Buys S, Daly MB, Dempsey PJ, Farrar WB, Fleming I, Garber JE, Harris RE, Heerdt AS, Helvie M, Huff JG, Khakpour N, Khan SA, Krontiras H, Lyman G, Rafferty E, Shaw S, Smith ML, Tsangaris TN, Williams C, Yankeelov T (2009). NCCN clinical practice guidelines in oncology: breast cancer screening and diagnosis. J Natl Compr Canc Netw.

[3] Biesecker BB (2001). Goals of genetic counseling. Clin Genet.

[4] Hall MJ, Reid JE, Burbidge LA, Pruss D, Deffenbaugh AM, Frye C, Wenstrup RJ, Ward BE, Scholl TA, Noll WW (2009). BRCA1 and BRCA2 mutations in women of different ethnicities undergoing testing for hereditary breast-ovarian cancer. Cancer.

[5] Armstrong K, Weber B, Stopfer J, Calzone K, Putt M, Coyne J, Schwartz JS (2003). Early use of clinical BRCA1/2 testing: associations with race and breast cancer risk. Am J Med Genet A.

[6] Armstrong K, Micco E, Carney A, Stopfer J, Putt M (2005). Racial differences in the use of BRCA1/2 testing among women with a family history of breast or ovarian cancer. JAMA.

[7] Hall MJ, Olopade OI (2006). Disparities in genetic testing: thinking outside the BRCA box. J Clin Oncol.

[8] Levy DE, Byfield SD, Comstock CB, Garber JE, Syngal S, Crown WH, Shields AE (2011). Underutilization of BRCA1/2 testing to guide breast cancer treatment: black and Hispanic women particularly at risk. Genet Med.

[9] Sussner KM, Edwards T, Villagra C, Rodriguez MC, Thompson HS, Jandorf L, Valdimarsdottir HB (2014). BRCA genetic counseling among at-risk Latinas in New York City: new beliefs shape new generation. J Genet Couns.

[10] Forman AD, Hall MJ (2009). Influence of race/ethnicity on genetic counseling and testing for hereditary breast and ovarian cancer. Breast J.

[11] van Riel E, van Dulmen S, Ausems MG (2012). Who is being referred to cancer genetic counseling? Characteristics of counselees and their referral. J Community Genet.

[12] CBS. Bevolking; generatie, geslacht, leeftijd en herkomstgroepering, 1 januari. 2014. Centraal Bureau voor de Statistiek Den Haag/Heerlen

[13] Dagevos J, Gijsberts M, Kappelhof J, Vervoort M (2007). Survey integratie minderheden 2006.

[14] Arnold M, Aarts MJ, Siesling S, Aa M, Visser O, Coebergh JW (2013). Diverging breast and stomach cancer incidence and survival in migrants in The Netherlands, 1996–2009. Acta Oncol.

[15] Arnold M, Razum O, Coebergh JW (2010). Cancer risk diversity in non-western migrants to Europe: an overview of the literature. Eur J Cancer.

[16] Egeli U, Cecener G, Tunca B, Tasdelen I (2006). Novel germline BRCA1 and BRCA2 mutations in Turkish women with breast and/or ovarian cancer and their relatives. Cancer Invest.

[17] Laarabi FZ, Jaouad IC, Ouldim K, Aboussair N, Jalil A, Gueddari BE, Benjaafar N, Sefiani A (2011). Genetic testing and first presymptomatic diagnosis in Moroccan families at high risk for breast/ovarian cancer. Oncol Lett.

[18] Tazzite A, Jouhadi H, Nadifi S, Aretini P, Falaschi E, Collavoli A, Benider A, Caligo MA (2012). BRCA1 and BRCA2 germline mutations in Moroccan breast/ovarian cancer families: novel mutations and unclassified variants. Gynecol Oncol.

[19] Laraqui A, Uhrhammer N, Lahlou-Amine I, El RH, El BJ, Dehayni M, Moussaoui RD, Ichou M, Sbitti Y, Al BA, Amzazi S, Bignon YJ (2013). Mutation screening of the BRCA1 gene in early onset and familial breast/ovarian cancer in Moroccan population. Int J Med Sci.

[20] Slaoui M, Razine R, Ibrahimi A, Attaleb M, Mzibri ME, Amrani M (2014). Breast cancer in Morocco: a literature review. Asian Pac J Cancer Prev.

[21] Bou KR (2013). Attitudes, beliefs and perceptions regarding truth disclosure of cancer-related information in the Middle East: a review. Palliat Support Care.

[22] Razum O, Zeeb H, Akgun S (2001). How useful is a name-based algorithm in health research among Turkish migrants in Germany?. Trop Med Int Health.

[23] Spallek J, Kaatsch P, Spix C, Ulusoy N, Zeeb H, Razum O (2006). Name-based identification of cases of Turkish origin in the childhood cancer registry in Mainz. Gesundheitswesen.

[24] Spallek J, Arnold M, Hentschel S, Razum O (2009). Cancer incidence rate ratios of Turkish immigrants in Hamburg, Germany: a registry based study. Cancer Epidemiol.

[25] Hoopman R, Terwee CB, Muller MJ, Ory FG, Aaronson NK (2009). Methodological challenges in quality of life research among Turkish and Moroccan ethnic minority cancer patients: translation, recruitment and ethical issues. Ethn Health.

[26] Stichting Opsporing Erfelijke Tumoren and Vereniging Klinische Genetica Nederland, Werkgroep KlinischeOncogenetica. Erfelijke Tumoren: Richtlijnen voor diagnostiek en preventie. 2005

[27] Rich EC, Burke W, Heaton CJ, Haga S, Pinsky L, Short MP, Acheson L (2004). Reconsidering the family history in primary care. J Gen Intern Med.

[28] Aarts MJ (2012) Socioeconomic determinants of cancer risk, detection, and outcome in the Netherlands since 1990. Dissertation, Mieke Aarts

[29] IKNL (2014) Incidentie borstkanker 2012

[30] Ozmen V, Ozcinar B, Karanlik H, Cabioglu N, Tukenmez M, Disci R, Ozmen T, Igci A, Muslumanoglu M, Kecer M, Soran A (2009). Breast cancer risk factors in Turkish women–a University Hospital based nested case control study. World J Surg Oncol.

[31] Sezer H, Yilmaz M, Gurler H, Koyuncu A (2011). Breast cancer risk factors in Turkey: a hospital-based case-control study. Asian Pac J Cancer Prev.

[32] Le registre des Cancers de la Region du grand Casablanca 2005-2006-2007. Edition 2012

[33] Laamiri FZ, Otmani A, Ahid S, Barkat A (2013). Lipid profile among Moroccan overweight women and breast cancer: a case-control study. Int J Gen Med.

[34] CBS. Statline, Bevolking; geslacht, leeftijd, nationaliteit en regio, 1 januari (jaren 2007 en 2012). 2014. Centraal Bureau voor de Statistiek, Den Haag/Heerlen

[35] Lucassen A, Laarman C (2009). Immigration, intermarriage and the changing face of Europe in the post war period. Hist Fam.

[36] Graeser MK, Engel C, Rhiem K, Gadzicki D, Bick U, Kast K, Froster UG, Schlehe B, Bechtold A, Arnold N, Preisler-Adams S, Nestle-Kraemling C, Zaino M, Loeffler M, Kiechle M, Meindl A, Varga D, Schmutzler RK (2009). Contralateral breast cancer risk in BRCA1 and BRCA2 mutation carriers. J Clin Oncol.

[37] Malone KE, Begg CB, Haile RW, Borg A, Concannon P, Tellhed L, Xue S, Teraoka S, Bernstein L, Capanu M, Reiner AS, Riedel ER, Thomas DC, Mellemkjaer L, Lynch CF, Boice JD, nton-Culver H, Bernstein JL (2010). Population-based study of the risk of second primary contralateral breast cancer associated with carrying a mutation in BRCA1 or BRCA2. J Clin Oncol.

[38] van der Kolk DM, de Bock GH, Leegte BK, Schaapveld M, Mourits MJ, de Vries J, van der Hout AH, Oosterwijk JC (2010). Penetrance of breast cancer, ovarian cancer and contralateral breast cancer in BRCA1 and BRCA2 families: high cancer incidence at older age. Breast Cancer Res Treat.

